# Enhanced YOLO12 with spatial pyramid pooling for real-time cotton insect detection

**DOI:** 10.1038/s41598-026-35747-4

**Published:** 2026-02-03

**Authors:** Dina Saif, Heba Askr, Amany M. Sarhan, Aboul Ella Hassanien

**Affiliations:** 1 Faculty of Artificial Intelligence, Delta University for Science and Technology, Gamasa, 35712 Egypt; 2 Faculty of Computers and Artificial Intelligence, University of Sadat City, Menoufia, Egypt; 3 Computer and Control Engineering Department, Faculty of Engineering, Tanta University, Tanta, Egypt; 4 Faculty of Engineering, Elsewedy University of Technology, 10th of Ramadan, Egypt; 5 Faculty of Computers and Artificial Intelligence, Cairo University, Cairo, Egypt; 6 Scientific Research School of Egypt (SRSEG), Giza, Egypt

**Keywords:** Cotton insect detection, YOLO12, Deep learning, Insect management, Object detection, Precision agriculture, Sustainability, Ecology, Ecology, Engineering, Mathematics and computing

## Abstract

Effective insect detection is crucial for sustainable cotton production, yet traditional monitoring methods remain labor-intensive, inefficient, and environmentally detrimental. This study introduces Enhanced YOLO12, a novel deep learning architecture for real-time cotton insect detection. Building on the YOLO12 framework, the proposed model integrates an optimized Spatial Pyramid Pooling (SPP) module and attention-based feature extraction to improve detection accuracy while maintaining computational efficiency. To ensure robustness, we developed and evaluated multiple baseline models (standard YOLO11 and YOLO12) and custom architectures (YOLO12_Fusion, YOLO11-BRA-Net, YOLO11_CBAM, and Enhanced Hybrid YOLO12). According to the conducted experiments, Enhanced Hybrid YOLO12 achieved the best performance, achieving 0.942, 0.876, 0.945, and 0.735 in precision, recall, mAP50 and mAP50-95, respectively. It significantly outstands the results of the standard YOLO12 (0.925, 0.848, of 0.913, and 0.662). These results demonstrate that Enhanced Hybrid YOLO12 can be considered as a state-of-the-art framework for precision agriculture, with its high detection accuracy and real-time capability. Therefore, they encourage this deep learning model in pest management applications.

## Introduction

Cotton is known to be the most important crops and a primary source of fibre for the textile in many countries around the world. Cotton is the main fabric of clothing, home textiles, and many other industrial products due to its versatility, affordability, and comfort. In addition, cotton seeds are utilized for oil extraction which is then used in numerous industries, including food, cosmetics, and industrial purposes. Millions of workers are employed in the cotton business including cultivation, harvesting, processing, manufacture, and retail. It is essential in developing countries because it supports auxiliary industries like oil, animal feed, and biofuels and generates revenue for smallholder farmers^1^.

Over 110 countries produce cotton, with China, India, the US, Egypt, and Pakistan being the top producers. Exports of cotton boost income, encourage rural development, and lessen poverty^2^. However, cotton breeding faces difficulties due to pests, which raises production costs and reduces profits. Maintaining quality requires effective pest management. Traditional techniques are costly and harmful to the environment. To reduce computing costs and guarantee efficiency, these needs require the help of an automated tool that uses computer technology^1^.

Advances in technology, especially computer vision and machine learning (ML) in many fields^2–5^ especially precision agriculture^6^ have caused the pest control revolution, allowing automated pest detection and classification of cotton insects to rely on broad-spectrum pesticides and support sustainable procedures^7,8^. Previous studies on the use of deep learning models (DL) to identify cotton pests were limited, despite their significance and consequences in the industry. For example, Alves et al.^9^ invented the classification method for 15 categories of pests using a deep residual learning model and achieved F1-score of 0.98. On the other hand, Bai et al.^10^ introduced the MSP2P, a point frame that alleviates the limitation of boxes and class imbalances, increasing the accuracy of detection and insect enumeration. R. LI et al.^11^ presented CFNET-VOVGCSP-LSKNET-JOLOV8S, a model for identifying pests and diseases with impressive accuracy. Yang et al.^12^ introduced SRNET-YOLO to detect small pests and exceeded the current YOLO models by 32.2%. Liu et al.^13^ used visual transformers (VIT) after enlargement of the contrast to improve insect identification, while Qiu et al.^14^ introduced a SpimNet, a network using more exhausts for the extraction and classification of functions.

Although this progress allows the detection and classification of cotton insects, there are many gaps related to current literature. One of the largest gaps concerns the lack of data files that are publicly available, diversified and good quality. Many existing data sets are either small or skewed, creating an imbalance that limits the applicability of DL models in practical agricultural environments. This makes it difficult to accurate insect recognition under different conditions in the field and prevents the identification of the real-time solution. In addition, the efficiency of speed and memory is decisive for processing an image of the field for real -time derivation, which will reduce the scanning time. This research focuses on the DL application for identifying and classification of cotton insects. Our goal is to create a comprehensive monitoring system for cotton insects, which allows automated insect control by means of image analysis, DL algorithms and other AI tools. By improving insect monitoring and reducing chemical intervention, agricultural operations can be optimized, and both the environment and human health can be improved. To the best of our knowledge, this is an innovative work that utilizes YOLO12. Additionally, we use spatial pyramid pooling to further improve its performance. The goal of this work is better feature extraction in applications containing small objects, like the one at hand. The following points encapsulate the research’s contribution:

1. Addressing the shortage of deep learning (DL)-based frameworks for the detection of cotton insects, which is essential for precise agriculture. It creates innovative methods and a strong foundation for next studies on intelligent, real-time insect monitoring systems.
2. Making use of a recently released open-access dataset for cotton insects that comprises 3,225 high-resolution photos of 13 different insect types, guaranteeing reliable and varied training data for generalization.
3. Creating and testing a number of YOLO-based model architectures for the identification and detection of cotton insects, including base YOLO models (YOLO11 and YOLO12) and improved models that use advanced feature extraction techniques and complex attention mechanisms.
4. Extensive testing of four improved versions (YOLO12_Fusion, YOLO11-BRA-Net, YOLO11_CBAM, and Enhanced Hybrid YOLO12), alongside baseline architectures (YOLO11 and YOLO12), demonstrated that the Enhanced YOLO12 model is the most successful, achieving superior localization precision, fewer false detections, and increased robustness in complex agricultural environments. Moreover, its real-time performance makes it suitable for field deployment, directly supporting early insect identification, reducing pesticide use, and promoting sustainable cotton production.

The rest of this research is structured as follows. Literature on cotton diseases and the detection and classification of cotton insects using DL models is summarized in “Related work”. The proposed model is given in “The proposed cotton insect detection model”. The analysis and discussion of the results are shown in “Result analysis and discussion”. “Conclusion and future work” concludes the paper and gives suggestions for future work.

## Related work

Despite the existence of numerous DL models to detect and classify plant diseases, a limited number of articles dealing with DL applications in the detection of cotton insects suggest a gap in the existing literature. The importance of cotton as a key economic crop underlines the need for more sophisticated and specialized detection models due to a lack of research in this area. This section provides a comprehensive overview of contemporary literature on diseases of plants, cotton diseases, and the detection and categorization of cotton insects with DL models, along with limitations in the literature. Table 1 summarizes the reviewed literature.

### Detection and classification of plant diseases

In the detection and classification of plant diseases, numerous studies use DL to detect and classify plants, including the work of S. Sharma and M. Vardhan^15^, who introduced a new method for identifying medicinal plants, emphasizing their cost efficiency and minimal side effects. The network of local and global features based on attention (AELGNET) uses adaptive levelling of the histogram with contrast to the extraction of prominent properties and overcomes 14 existing approaches for accurate identification in medical and industrial applications. D. Chen et al.^16^ introduced a model with a small Jolov8-MDN, which aims to increase the detection of the disease on a small scale in a commercially significant crop affected by wasting. The traditional DL models face challenges in accurately identifying cases of diseases on a small scale. The proposed model deals with this restriction on the improvement of the localization of small targets of the fruit of the passion, increasing mAP50, accuracy, and evoking in reducing the model parameters and using memory. The finding promotes effective detection of the disease in the posthumous fruit of the passion and facilitates real-time sorting processes.

A. Dheeraj and S. Chand^17^ suggested the use of the ECENET model built into the improved convolution block attention (CBAM) for agricultural crop and corn classification. The model components include a deep neural network with a thin client and CAM and SAM settings. Compared to Densenet121, Inctectresnetv2, Resnet50v2 and Xcectionnet, this model achieved a total accuracy of 99.92% recognition using smaller parameters. Agricultural robots can use this model to perform real-time weed and work well for categorization on mobile devices. P.

Chavan et al.^18^ introduced a hybrid architecture for agriculture, solving problems related to the composition of soil, climate, and pathogens. The model uses DL methodology and image processing to detect diseases and the crop of leaves. The model consists of two phases: prediction of crops and identification of leaf disease. The input image is pre-processed, and functions such as shape, colour, and texture are extracted. The model, implemented in Python, has a higher detection accuracy of 0.982 and F-measure of about 0.956, which shows excellent performance compared to other techniques.

### Detection and classification of cotton diseases

In the detection and classification of cotton diseases, several studies are using DL to detect and classify cotton diseases. A. Pavate et al.^19^ used an architecture of neural networks of efficient YOLO to develop a prediction model to identify cotton diseases in cotton plants. The model helps farmers to use pesticides to treat the disease. The model responds to inputs from different sources and monitors different results. The EfficientNetB4 model achieved 100% accuracy for healthy leaves and mild leaves, while the YOLOV4 achieved 96% precision, recall, and 0.70 precision.

- S. Madhu and V. Ravisankar^20^ designed a DL model based on YOLO to identify leaf disease in the evaluation of the health of cotton plants. The model integrates image processing methodologies to increase the accuracy of the disease identification. YOLOv5 overcomes VGG16 and Resnet50 in accuracy and effectively detects and classifies various diseases affecting cotton plants, improving monitoring and health management in cotton agriculture. H. Askr et al.^6^ developed a DL system to predict disease in cotton plants using explained artificial intelligence and optimization of a Gray-based wolf. The system uses images, preliminary processes, and uses the copula entropy to select functions and achieves an accuracy of the classification of 99%.
- R. Kumar et al.^21^ developed a ML-based system to detect disease in cotton plants using images of leaves, increase agriculture production and yield, and emphasize the need for further research. C. Singh et al.^22^ developed a hybrid methodology of DL, Bert-Resnet-PSO, to identify diseases of cotton plants, achieving 98.5% accuracy, 98.2% precision, and 98.7% recall on the Plant Village data set, promising to alleviate crop loss and effective management.

### Detection of cotton insects

In the detection of cotton insects, Alves et al.^9^ developed the classification methodology for the main cotton pests (15 categories of pests) using an innovative data set and a deep residual teaching framework. The ResNet34 has an accuracy of 0.98, which makes it possible to detect pests and implement environmentally friendly and economically feasible methods. Bai et al.^10^ introduced a new framework for the identification and listing of multi-type insects called MSP2P, which overcomes the limitation of border boxes. The method uses a light identification network of objects to predict the regression of points and a point design to increase sensitivity to fine insect properties. MSP2P optimizes subsequent processing procedures and solves problems with class imbalance, achieving optimal results in localization and counting metrics on the natural cotton data set on the natural scene and YST data set. Li et al.^8^ developed a new model, CFNET-VOVGCSP-LSKNET-JOLOV8S, which increases conventional pest detection methods in cotton leaves, has an accuracy of 89.9%, and an average accuracy of 93.7%.

Yang et al.^12^ have developed SRNet-YOLO, a framework for identifying minute and extremely small pests in natural cotton fields. It uses YOLOv8 extraction, reconstruction of FM-SR maps, and BiFormef fusion, which is a 32.2% improvement in detection performance. Liu et al.^13^ developed a visual transformer model for improved identification of agricultural pests, increased accuracy through the merger of spatial elements, improved contrast, and conventional network graphs. Qiu et al.^14^ developed a SpemNet, a network of classification of images for detecting cotton pests and diseases. The network uses a more effective measure of attention and stacking modules to insert modules to improve local extraction and integration of information.

Insect identification research faces challenges due to restrictions and gaps, including a lack of high-quality data sets for the training of robust DL models, which are often small, imbalanced, and are not fully agricultural controlled environments in the real world. This limits the generalization capacity of DL models when applied to different conditions in the field, where factors such as lighting changes, clutter in the background, and a variety of pests significantly affect detection. In addition, many data files focus on specific types of pests and leave gaps in the comprehensive identification of multiple yards. Without large, diverse, and well-annotated data sets, DL models seek to achieve high accuracy and adaptability in the detection of cotton insects under various environmental and geographical conditions.

### Detection of small objects

Ciccone et al.^23^ Ciccone et al. presented a thorough approach that makes use of a two-stage transfer learning strategy: pretraining on the VisDrone dataset for general aerial object detection, followed by fine-tuning on the Heridal dataset specifically designed for search and rescue situations. To increase sensitivity to small, sparsely located human figures while preserving computational efficiency, they investigated architectural changes like improved feature fusion (FPN, BiFPN, PB-FPN), extra detection heads, and modules like CBAM, Transformers, and deconvolution. With real-time inference capability on embedded hardware (Jetson Nano), their top-performing configuration, YOLOv5s-PBfpn-Deconv, obtained a mAP@50 of 0.802 on the Heridal dataset. Additionally, they demonstrated that lightweight, interpretable AI systems can be successfully deployed on UAVs for civil protection search and rescue missions by validating robustness across various flight altitudes and improving interpretability using EigenCAM.

Giri^24^ improves the YOLOv8 model’s architecture by adding a Squeeze-and-Excitation (SE) block, advanced multi-scale training, and data augmentation methods that help it better capture the fine details of small objects. The model was tested on the PASCAL VOC 2012 dataset and did better than the baseline YOLOv8, with a precision of 1.0 (up from 6%) and a mean Average Precision (mAP) of 0.79. By showing that specific architectural changes can improve YOLO’s capacity to identify small objects, the work lays a solid platform for future research in this field.

AgarwoodNet, a robust but computationally economical substitute for heavy deep learning networks that require a lot of resources and take a long time to train, is presented by Shafik et al.^23^. The Agarwood Pest and Disease Dataset (APDD) is a newly curated dataset that they utilize to train and test their model. 5,472 annotated photos of agarwood leaves from Turkey and Brunei, categorized into 14 classes, make up this collection. The study demonstrates how lightweight AI models may solve issues like scalability, training efficiency, and adaptability to enhance plant health monitoring and ensure there is enough food for everyone.

Zhang et al.^25^ examines the difficulties in identifying minor disease characteristics and pests in cistanche plants cultivated in Inner Mongolia. The suggested model combines low-level details with high-level semantic features in a way that changes over time. This makes it possible to find diseases even when the background is busy and the symptoms are hard to see. The results of the experiments show that the system works very well, with an average accuracy of 0.93, a precision of 0.95, a recall of 0.92, and mAP@50 and mAP@75 scores of 0.92 and 0.90, which is better than traditional self-attention mechanisms and CBAM modules.

## The proposed cotton insect detection model

**Fig. 1:**
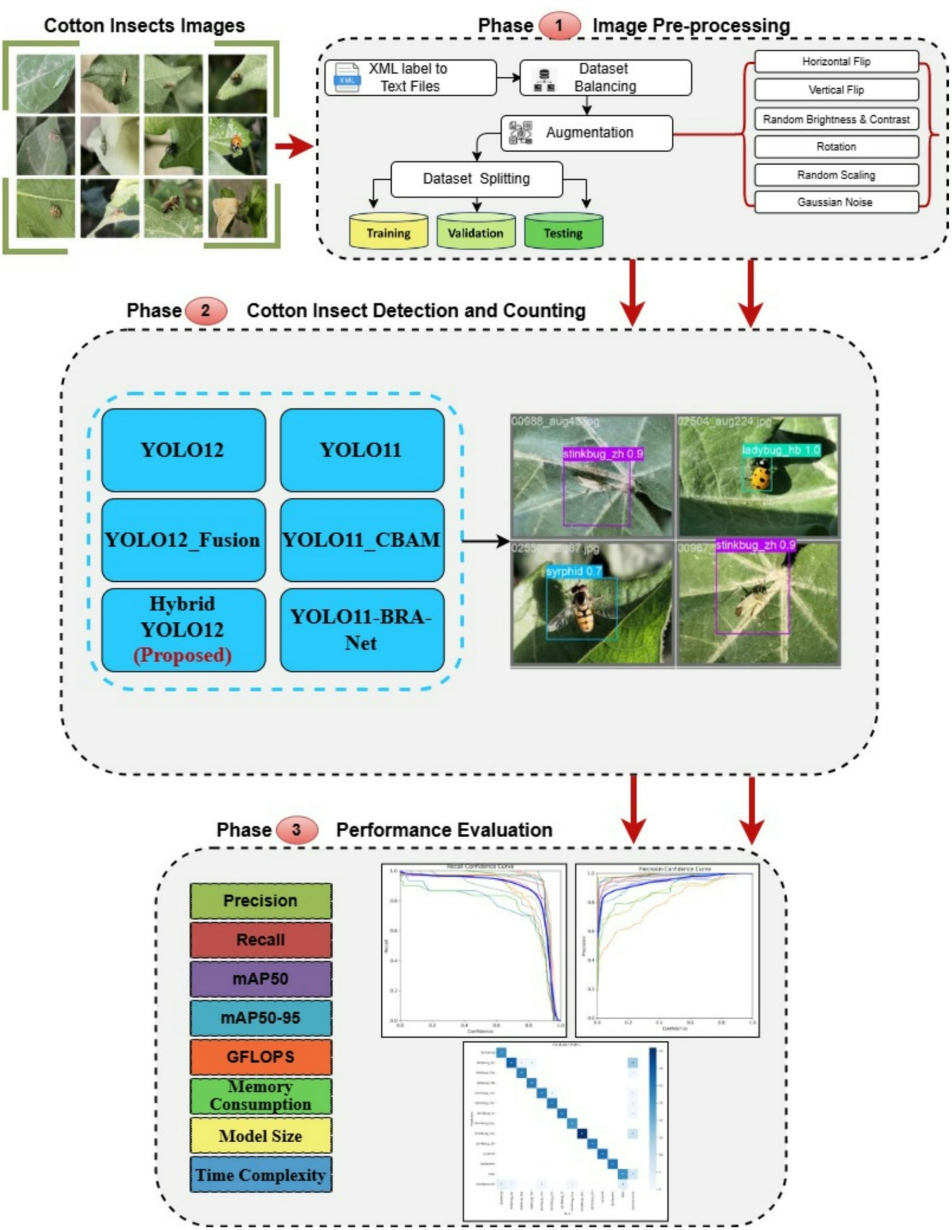
The proposed model for the cotton insects’ detection problem.

This section introduces the methodology used in developing the proposed Enhanced Hybrid YOLO12. This model is a new improved hybrid version of the recent standard YOLO12 deep learning model designed for cotton insect detection application. The main objective of this proposed model is to accurately classify the cotton insects. The proposed methodology is comprised of three main phases: dataset pre-processing, cotton insect detection, and model performance evaluation, as presented in Fig. 1. Each of these phases will be discussed in detail in the next subsections.

### Phase 1: dataset pre-processing

**Fig. 2:**
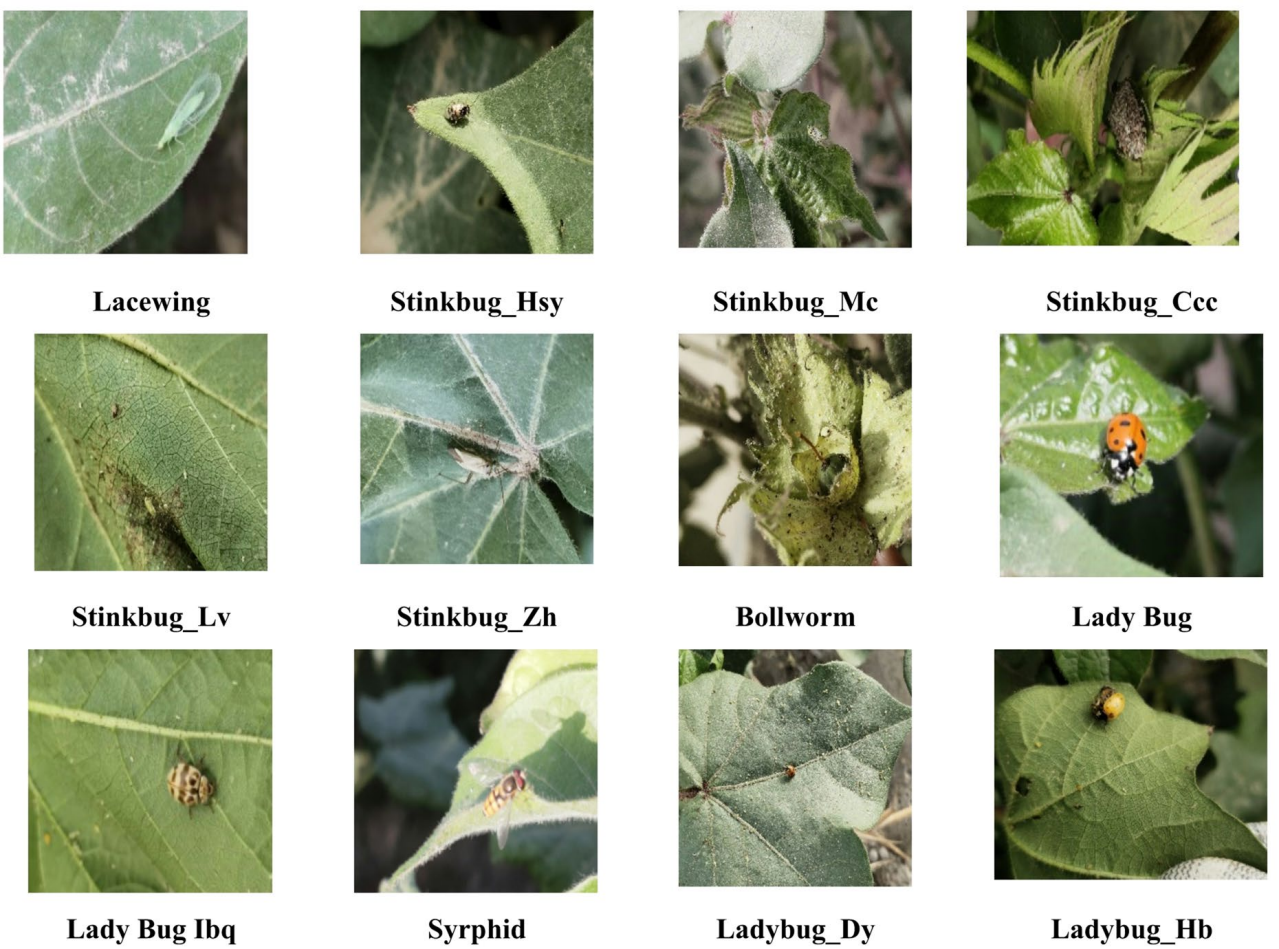
Samples from the cotton insect dataset (before image pre-processing).

This phase includes the preparation of input images for training using a number of image enhancement and augmentation techniques to increase the variability of datasets and improve the model’s generalization. Enhancement techniques include the conversion of annotation format and data balance. Augmentation techniques include flipping, rotation, and noise injection, which are applied to imitate real-world conditions and enhance the diversity of the appearance of insects. To demonstrate the efficiency of the proposed model in cotton recognition and pest recognition, we have implemented experiments using image data from a publicly available cotton data set. This data file is specially designed for insect detection in cotton fields and includes images of various common pests. It contains 13 classes and includes 3,225 high-quality images that capture their properties at different stages of growth and activity levels. Some samples of the predominant insects of the cotton field in the cotton data set are shown in Fig. 2.

In order to optimize the dataset for effective model training, a number of significant pre-processing enhancement are made as follows:

1. Annotation Format Conversion: To prepare a data set for training with the framework of YOLO object detection, we have converted annotation files from Pascal VOC (XML) to YOLO (TXT) format. Each original XML file contained information about metadata and objects, such as image dimensions, classes, and coordinates of border boxes (xmin, ymin, xmax, ymax). In the converting process, we first extracted the width and height of the image to normalize the coordinates of the border box. For each object, the class label was mapped to the integer ID, and the boundary box was converted to the YOLO format, which uses normalized values ​​for the center coordinates (X_CENER, Y_CENER) and the width and height of the boundary box. The resulting .txt file for each image contains one line on the object in the format: Class_id, x_center, y_center, width, and height. This step ensured compatibility with YOLO-based models that require annotations in this specific structure for training and derivation.
2. Balance of datasets: To prevent distortion, we edited the number of images per class. Classes exceeding 300 images have undergone down-raising to remove redundancy, while classes with less than 300 images were enlarged by data enlargement to increase representation.

**Table 1:** A summary of the current literature for DL in detecting and classification of cotton insects.

Ref.	Year	Model	Performance	Dataset	Number of images	Limitations
**Detection and classification of plant diseases**						
^15^	2025	AELGNet as new enhanced DL model.	Accuracy (99.71%), Precision (99.80%), Recall (99.75%) and F1 score (99.77%).	A dataset of Indian medicinal plants^26^	12,845 medicinal plants images.	The model is not generalized to novel, unobserved plant classifications, necessitating further examination.
^16^	2025	A YOLOv8-MDN-Tiny model.	Precision (91.5%), Recall (91.6%) and mAP50 (94.8%).	The dataset is developed by the authors in^16^	3630 medicinal plants images.	The model needs optimization and adjustment for its parameters to guarantee its efficacy in practical applications.
^17^	2025	Enhanced Convolutional Block Attention Module (CBAM) embedded EfficientNet model (ECENet) model.	Overall recognition accuracy (99.92%).	The corn weed dataset^27^	6000 crop images.	The model is not evaluated using a comprehensive image dataset encompassing a varied range of crop and species to enhance the model’s overall efficiency and complexity.
^28^	2025	Improved LinkNet + LeNet.	Accuracy (98.2%) and a F-measure (95.6%).	Plant Village dataset^29^	17 different classes in the dataset, including both healthy and sick samples from different crops (12655 images).	The increased value of the complexity of the proposed model.
^19^	2025	EfficientNet and YOLO neural network.	EfficientNetB4: Accuracy (100%) YOLOv4: Precision (96%), Recall (98.3%), mAP@0.5 (99.2%), and AP120.5:095 (0.70).	Collected dataset from Kaggle^30^	1340 color images with six classes.	Future enhancement can be considered as an exploration of additional data sources.
**Detection and classification of cotton diseases**						
^20^	2025	VGG16, ResNet50, and YOLOv5 models.	F1 score (99.21%, 99.21% and 98.88%) for VGG16, ResNet50 and YOLOv5, respectively.	Collected dataset from Kaggle^31^	5000 plant images.	While the proposed YOLO-based DL model demonstrates high accuracy in cotton plant leaf disease detection, future work should involve testing the model on a more diverse and comprehensive dataset that includes varying environmental conditions, lighting, and disease stages.
^6^	2024	Explainable ResNet50 model.	Accuracy (99.67%).	Collected dataset from Kaggle^33^	2400 images after balancing.	An opportunity exists to investigate alternative metaheuristic algorithms beyond GWO for disease detection. Additionally, incorporating Generative Adversarial Networks (GANs) could enhance the model’s predictive capabilities.
^21^	2024	Ensemble RF and Decision Tree model.	Precision (94.5%).	Diverse dataset of cotton leaf images for disease classification^30^	3000 crop images.	The limitations are primarily the dependency on diverse, high-quality datasets for rare or evolving diseases and the challenge of balancing interpretability and performance in the hybrid model’s decision-making process.
^22^	2025	A hybrid DL approach.	Accuracy (98.5%), Precision (98.2%), and Recall (98.7%).	Plant Village dataset^29^	4,000 high resolution images of cotton leaves.	The need for optimization of certain features and adaptability to varying environmental conditions.
**Modern andsmall object detection**						
^23^	2025	YOLOv5s-PBfpn-Deconv	mAP@50 of 0.802	VisDrone^34^	10,209 images and 261,908 video frames.	The paper is limited by the small size and narrow scope of the Heridal dataset, controlled experimental conditions, and hardware constraints that restrict scalability and generalization to diverse real-world search and rescue scenarios.
^24^	2025	SO-YOLOv8	mAP of 0.79	PASCAL VOC 2012 dataset^35^	11,000 images with annotated objects across 20 categories.	Despite improvements, the model was only tested on the VOC 2012 dataset, limiting its real-world applicability.
^36^	2025	AgarwoodNet	classification accuracy of about 97%	Agarwood Pest and Disease Dataset^37^	5,472 images of agarwood leaves.	Limitations include reliance on a single curated dataset, restricted environmental diversity, and untested scalability across broader agricultural contexts.
^25^	2025	Transformer-Based Detection Network with Bridging Attention (TBDA-Net).	mAP@50: 0.92	Cistanche Pest and Disease Dataset (CPDD)^25^	4,800 annotated images across diverse pest and disease categories for distance plants.	The paper is constrained by its dependence on a region-specific distance dataset, regulated experimental conditions, and unverified scalability across various crops, environments, and practical agricultural applications.
**Detection of cotton insects**						
^9^	2020	Fine-tuned Deep residual learning model ResNet34*.	Accuracy (98.1%).	Collected dataset in Brazil^38^	1,600 images from 15 classes and after augmentation become 9,920 images.	Limitations include challenges like lighting, background clutter, and pest variability. The dataset lacks representation of diverse environmental conditions and requires expansion for better pest variability capture.
^10^	2024	The YOLOv7-tiny object detection framework and LAHead are used for feature extraction and insect classification, utilizing SmoothL1 Loss and Focal Loss to prevent class imbalance.	For YST dataset: Precision (91.7%), Recall (92.6%), and F1 score (91.3%). For NSC dataset: Precision (85.8%), Recall (79.8%), and F1 score (81.9%).	Self-constructed NSC dataset in China^10^ and Publicly accessible YST dataset^39^ for validation	369 images from 9 insect species where each images contains insects captured in a static environment using Scout boxes.	The MS-P2P model, based on yellow sticky trap datasets, has shown lower performance in complex agricultural settings and is less robust than the YOLO series models.
^11^	2024	CFNet-VoVGCSP-LSKNet-YOLOv8s.	Precision (89.9%), Recall (90.7%), and mAP@0.5 (93.7%).	Collected dataset from six freely accessible cotton pest and disease databases on Kaggle^40^	4,703 images for pests and diseases and after augmentation become 5,927 images.	The proposed model for detecting cotton leaf spot disease can be improved by incorporating multi-channel scale attention mechanisms and refining bounding box optimization.
^12^	2024	SRNet-YOLO.	mAP (92.4%) for “tiny pests” and mAP (57%) for “very tiny pests”.	Cotton-Yellow-Sticky-2023 dataset	3712 images from 5 pests including tiny and very tiny pests.	Although the detection performance for small pests is satisfactory, the accuracy for very tiny pests remains low at only 57%, highlighting the need for further improvements.
^13^	2024	A Vision Transformer-based model with spatial feature fusion and contrast enhancement (SFCE-VT).	Precision (77.94%) for IP102 dataset, (92.69%) for CUB-200-2011 dataset and (84.65%) for A-pests dataset.	CUB-200-2011^42^, IP102^43^, wolfberry pest^44^ and cotton pest ^45^	9231 images for agriculture pests (A pests) includes 3225 images for cotton pests.	While the current model achieves competitive results, integrating it with larger, more powerful models could further enhance recognition accuracy.
^14^	2024	A novel cotton pest and disease classification network called SpemNet based on efficient multi-scale attention and stacking patch embedding.	Precision (99.03%) for cotton insect identification and (94.87%) for IP102 leaves disease.	Cotton Insect dataset^45^ and IP102^43^	5314 images for cotton insects from 13 classes.	The model needs to be tested in real-world field images. SpemNet’s performance in multi-output classification tasks is also limited, as it may bias towards the most prominent feature.

3. Advanced data augmentation: We used multiple augmentation techniques (as shown in Table 2) to improve the model generalization including:

- Horizontal and vertical overturning: mirroring images to introduce variability.
- Adjusting brightness and contrast: increasing the diversity of the image.
- Rotation and random scaling: introduction of different perspectives and size changes.
- Gaussian noise injection: Improving the robustness of the model simulations of the real-world image.

**Table 2:** Configuration parameters for dataset pre-processing.

Technique	Parameters
Horizontal flip	P (Probability) = 0.5.
Vertical flip	*P* = 0.2.
Random brightness and contrast	Brightness limit (Range for random brightness adjustment, default: 0.2), Contrast limit (Range for random contrast adjustment, default: 0.2).
Rotation	P (Probability) = 0.3.
Random scaling	Scale limit = 0.2, *P* = 0.3.
Gaussian noise	*P* = 0.2.

**Fig. 3:**
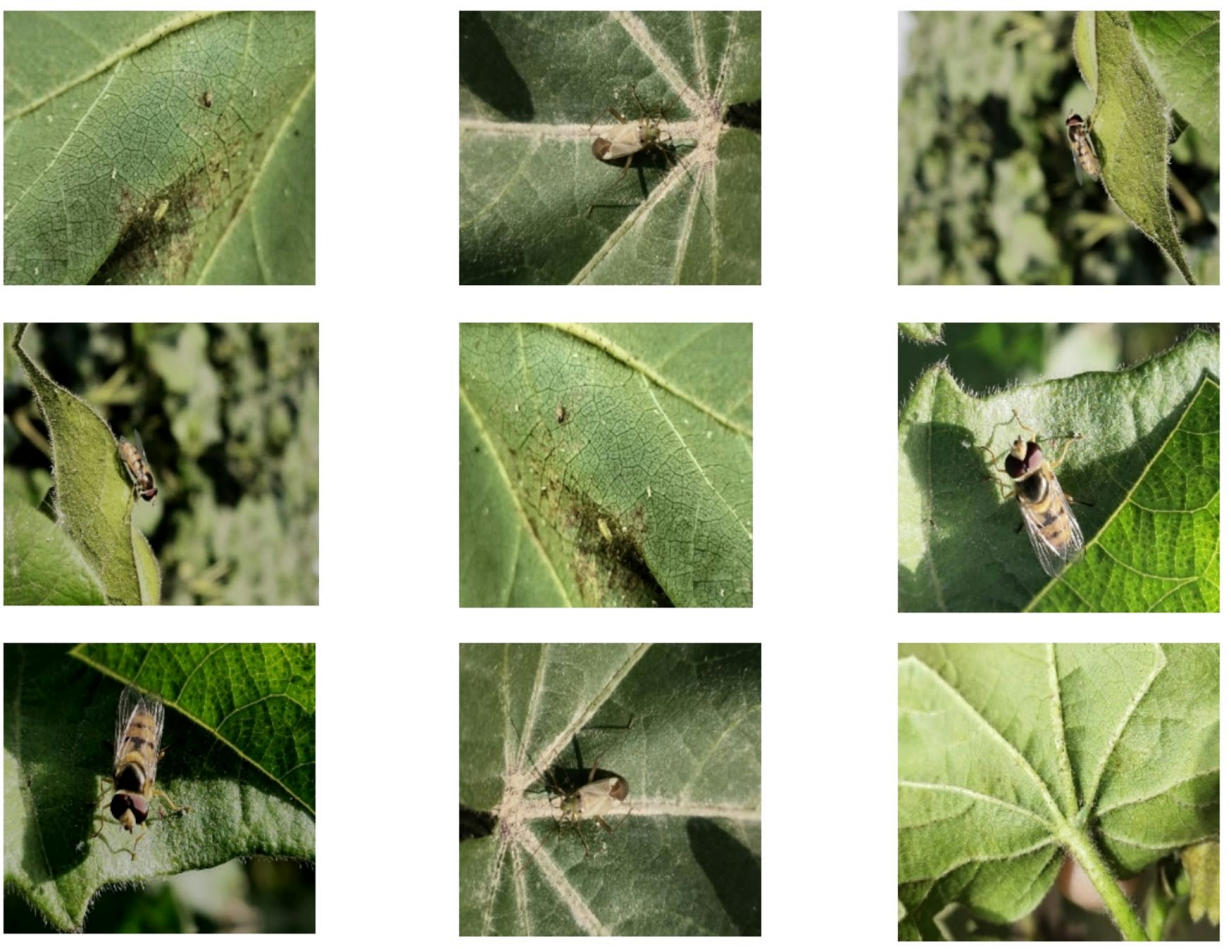
Samples from the cotton insect dataset (after image pre-processing).

The dataset is divided into 70% training, 10% verification, and 20% testing, which ensures a well-balanced distribution for efficient model evaluation. These steps prior to processing significantly increase data quality, reduce distortion, and strengthen the ability of the model to precisely detect and classify cotton insects in real-world scenarios. The visual representation of the processed data file is shown in Fig. 3.

### Phase 2: cotton insect detection

**Fig. 4:**
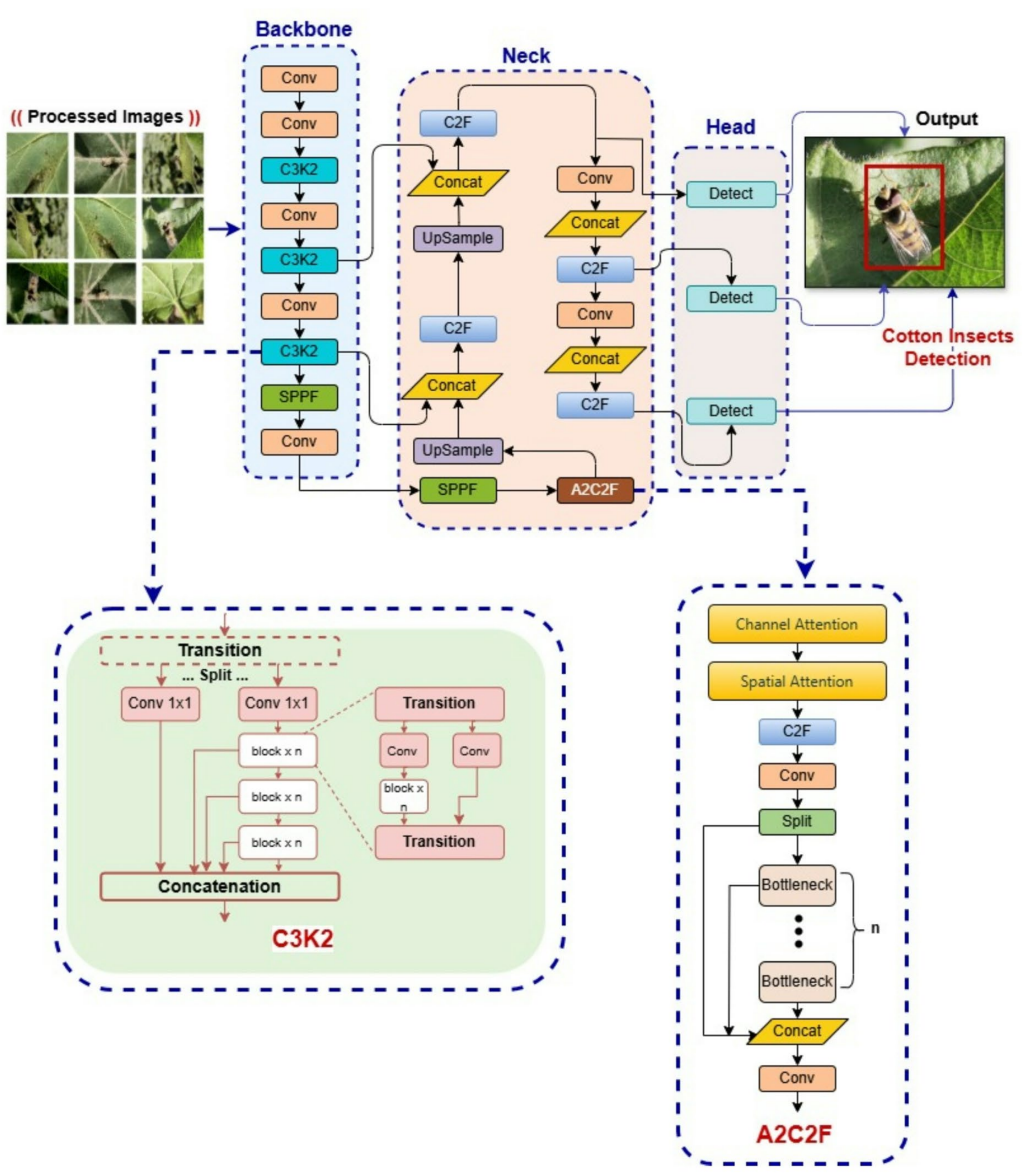
Architecture of the proposed enhanced hybrid YOLO12 model.

This phase presents the proposed enhanced hybrid YOLO12 model for the cotton insect detection problem as presented in Fig. 4. YOLO12 as the recent DL model for object detection is selected over alternative DL detection models because of its exceptional real-time detection capabilities, high precision in small objects recognition, and efficiency in managing extensive datasets with minimum computational costs. In addition to its capacity to harmonize speed and accuracy makes it optimal for agricultural applications where rapid and accurate insect detection is essential for efficient pest management^46^.

In this paper, the proposed improved version of YOLO12, which is called Enhanced YOLO12 introduces key modifications to both the backbone and head of the standard YOLO12 model to improve feature extraction and fusion efficiency. The Enhanced Hybrid YOLOv12 model enhances object detection via an improved architecture that includes a backbone featuring C3K2 (Cross Stage Partial bottleneck with 3 convolutional layers) blocks which belongs to YOLO7 and SPPF (Spatial Pyramid Pooling Fast) blocks which belongs to YOLO8 for feature extraction, a neck with C2F (Cross Stage Partial bottleneck with 2 convolutional layers) blocks and up sampling for multi-scale feature fusion, and an A2C2F (Attention Mechanism with C2F block) module that incorporates attention mechanisms to emphasize pertinent features, resulting in a decoupled head for accurate detection.

The integration of C3K2 blocks in the proposed Enhanced Hybrid YOLO12 architecture backbone (Fig. 4) enhances the feature extraction due to its transition-split-concatenation ability. The C3K2 block employs convolutional layers with dropout connections to efficiently capture both local and global information, dividing and processing feature maps in parallel prior to concatenation^47^.

Using the A2C2F block in the architecture of the proposed Enhanced Hybrid YOLO12 model (Fig. 4) aims to improve feature representation via including attention mechanisms and efficient convolutional processes. This block first performs channel and spatial attention, allowing the network to concentrate on the most important regions and features of the input, prior to transmitting the data through a series of convolutional layers and C2F modules^48^. The architecture necessitates the division of the feature map, processing the segments through bottleneck layers, and then concatenating the outputs which are integrated by convolution. The A2C2F block enhances the model’s capacity by integrating area-based attention (A2) with efficient feature aggregation (C2F), hence improving detection accuracy and processing efficiency inside the YOLO12 framework.

**Fig. 5:**
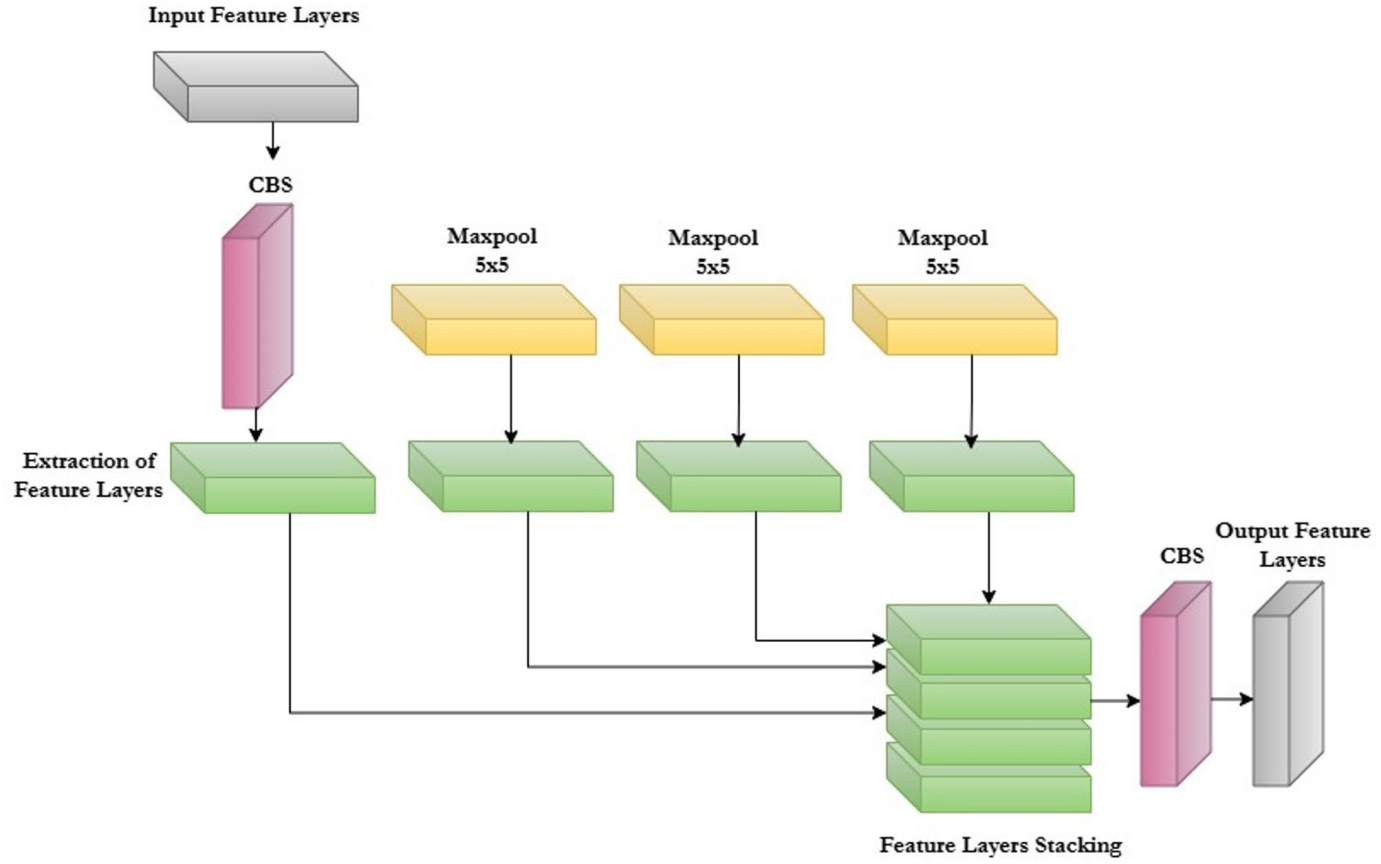
Architecture of the SPPF block.

The SPPF block (Fig. 5) is responsible for capturing more scales of spatial functions by combining operations in different core sizes. This increases the ability of the model to recognize objects of different sizes without significantly increased computing complexity.

The SPPF operation can be expressed in Eq. (1):

1 $$\:SPPF\left(X\right)\:=\:Concat\left(MaxPool\right(X,\:5),\:MaxPool(X,\:3),\:MaxPool(X,\:1\left)\right),$$

where $\:Concat$ indicates the operation of the classes applied along the size of the channel and $\:MaxPool$ $\:(X$, $\:k)$represents the maximum operation of the association applied to the map input function $\:X$ by the size of the c. This aggregation on multiple scales allows the network to better detect objects of different sizes enriching the representation of elements without significant increase in computing costs^49^.

To validate the performance of the Enhanced Hybrid YOLO12 model, we tested the cotton insect dataset on five other DL detection models. Two of these are the standard YOLO11 and YOLO12, while the remaining three are improved versions we developed based on YOLO11 and YOLO12: YOLO11_CBAM, YOLO11-BRA-Net, and YOLO12_Fusion, as detailed below.

#### Standard YOLO11

The first model is the standard YOLO11^50^, which has architecture structured into three key components: backbone, neck, and head. The backbone has the task of collecting critical properties using numerous convolutional layers and blocks of C3K2, which increase representation by shortcut connections. The neck increases the extracted features through sampling, clarification, and additional convolution layers to increase the fusion of functions on a larger scale. It prominently contains modules such as SPPF and C2PSA, which improve spatial and contextual consciousness. The head is detected by using many layers to “detect” on different scales to ensure precise localization and classification of objects. This architecture increases efficiency and accuracy, so it is suitable for insect detection in real-time in the agricultural environment.

#### YOLO11_CBAM

The second model is an improved variant of the standard YOLO11 called YOLO11_CBAM. This integration increases the original C3K module by integrating CBAM^51^, an effective attention mechanism that gradually implements the channel and spatial attention to emphasize significant aspects and at the same time reduce less relevant ones. The channel attention module captures inter-channel relations through a two-stage procedure, which includes the average operations of association and convolution, while the spatial attention module emphasizes spatial correlations through both average and maximum operations across spatial dimensions. CBAM integration into the C3K2 block increases the capacity of the model to concentrate on relevant features and therefore potentially expands the extraction of elements and the overall performance of the model.

#### YOLO11-BRA-Net

The third model is an enhanced variant of YOLO11 called YOLO11-BRA-Net. This model introduces a significant enhancement to the YOLO11 architecture by replacing the C3k2 modules with an state-of-the-art attention-augmented C3 block that incorporates Bi-Level Routing Attention (BRA)^52^. The original C3k2 module, which is an efficient variation of the CSP bottleneck, has the benefit of balancing the computational cost and representational power due to the stacked bottleneck or C3k layers. However, it has the problem of modelling long-range dependencies and adaptively prioritizing prominent features. To overcome this constraint, we will revise the C3 module to incorporate a BRA. This will ensure that it uses self-knowledge with more heads applied to a spatially flattened map of functions. This mechanism permits the network to perform dynamic routing of context elements, managed by interactions at the level of spatial and channels. The updated module uses a 1 × 1 convolution to reduce the dimensionality, includes the BRA process, and concludes with a 3 × 3 convolution to enhance the output. The architectural modification significantly increases the network’s ability to identify complex feature associations, thus enhancing object detection efficiency in real-life visual conditions.

#### Standard YOLO12

The fourth model, the YOLO12^53^ standard, which increases the extraction of the elements includes advanced attention mechanisms and improves more scale detection capabilities. It consists of three main ingredients: backbone, neck, and head. The backbone extracts comprehensive spatial information, the neck improves the representation of the functions on a larger scale, and the head uses more detection layers for accurate location and classification. This enhances accuracy and efficiency, so it is very suitable for use in agricultural insect identification and other real-time object detection applications.

#### YOLO12_Fusion

The fifth model is an enhanced version of YOLO12, named as YOLO12_Fusion. In this model, we modified the head shape by implementing a hierarchical fusion process that incrementally integrates multi-scale characteristics at various phases. The architecture retains the original backbone but introduces a four-group head strategy. First, high-level semantic features from P5 are Up-Sampling and fused with mid-level P4 features. Next, the result is again Up-Sampling and integrated with lower-level P3 features, preserving spatial details. To further increase the representation of the elements, the fusion branch re-aggregates the outputs from the earlier stages of the head and strengthens medium-level elements (P4). Finally, the next path of improvement is combined back to P5 using the functions of the backbone, which improves awareness of context and semantic depth.

### Phase 3: performance evaluation

To measure the performance of the proposed model for the cotton insect’s detection problem, several evaluation metrics are introduced in this paper such as precision, recall, F1 score, mAP50, mAP50-95, FLOPS, inference time, model size and memory consumption and they are illustrated in the following Eqs. (2), (3) and (4):

2 $$\:\mathrm{P}\mathrm{r}\mathrm{e}\mathrm{c}\mathrm{i}\mathrm{s}\mathrm{i}\mathrm{o}\mathrm{n}\:=\:\:\frac{\mathrm{T}\mathrm{P}}{(\mathrm{T}\mathrm{P}\:+\:\mathrm{F}\mathrm{P})},$$

3 $$\:\mathrm{R}\mathrm{e}\mathrm{c}\mathrm{a}\mathrm{l}\mathrm{l}\:=\:\:\:\frac{\mathrm{T}\mathrm{P}}{(\mathrm{T}\mathrm{P}\:+\:\mathrm{F}\mathrm{N})}\:,$$

4 $$\:\mathrm{F}1\:\mathrm{s}\mathrm{c}\mathrm{o}\mathrm{r}\mathrm{e}\:=\:2\:\times\:\:\:\:\frac{(\mathrm{P}\mathrm{r}\mathrm{e}\mathrm{c}\mathrm{i}\mathrm{s}\mathrm{i}\mathrm{o}\mathrm{n}\:\times\:\:\mathrm{R}\mathrm{e}\mathrm{c}\mathrm{a}\mathrm{l}\mathrm{l})}{(\mathrm{P}\mathrm{r}\mathrm{e}\mathrm{c}\mathrm{i}\mathrm{s}\mathrm{i}\mathrm{o}\mathrm{n}\:+\:\mathrm{R}\mathrm{e}\mathrm{c}\mathrm{a}\mathrm{l}\mathrm{l})}\:,$$

where TP (True Positive) denotes the count of samples correctly identified as positive by the model, TN (True Negative) signifies the count of samples accurately classified as negative, FP (False Positive) indicates the number of samples misclassified as positive despite being negative, and FN (False Negative) refers to the samples that are genuinely negative but incorrectly predicted as positive by the model.

FLOPS (Floating Point Operations) measure the number of arithmetic calculations performed by machine learning or a deep learning model during derivation, estimating calculation costs due to the capacity of the hardware processing. To determine the inference time, the ability to process hardware is evaluated, usually in fleets per second (FLOPS/s)^54^, as shown in Eq. (5). $\:L$ denotes the total count of layers within the model.

5 $$\:Total\:FLOPs=\sum\:_{i=1}^{L}FLOPs\:for\:layer\:i,$$

In machine learning and deep learning, the model’s size is crucial because it influences the memory and computational resources required for the model to function. Although more complete models with additional features could increase accuracy, they also require more storage and take longer to interpret data, which drives up computational costs. The total number of parameters in a neural network can be determined as in Eq. (6)^55^:

6 $$\:P=\sum\:_{l=1}^{L}({w}_{l}\times\:{h}_{l}\times\:{d}_{l}\times\:{n}_{l}),$$

where $\:{w}_{l}\:{h}_{l}\:and\:{d}_{l}$ are the width, height, and depth of a layer, and$\:\:{n}_{l}$​ is the number of filters or neurons.

In addition, memory usage is a critical component that affects the effectiveness of training and inference processes. Model size, batch size, and the accuracy of data representation (e.g., 32-bit or 16-bit floating integers) all affect the result. The total amount of memory required for a model includes storage for gradients, activations, and parameters during training. The memory utilization $\:M$ can be approximated as in Eq. (7)^56^:

7 $$\:M=P\times\:{S}_{p}+A\times\:{S}_{a}+G\times\:{S}_{g},$$

where $\:P$ is the number of parameters, $\:A$ is the number of activations, and $\:G$ is the number of gradients, while $\:{S}_{p}\:$​$\:{S}_{a}$ and $\:{S}_{g}\:$represent their respective storage sizes.

## Result analysis and discussion

The implementation of the proposed model in this paper was executed utilizing training in a CUDA environment using 15 GB GPU on Google COLAB PRO enabling efficient training and faster experimentation. Our training comprised 100 epochs on images of 640 × 640 resolution, a batch size of 8, and mixed precision (amp = True) for optimized memory usage.

### Performance evaluation of the proposed model versus state-of-the-art models

The efficacy of the proposed model (Hybrid YOLO12) is assessed through comparison with state-of-the-art models including the standard YOLO12, its improved variant YOLO 12_Fusion, the standard YOLO11, and two enhanced versions of YOLO11, namely YOLO11-BRA-Net and YOLO11_CBAM. The assessment measures considered are test precision, recall, mAP50, and mAP50-95.

Table 3 presents a comparative evaluation of six YOLO-based models, highlighting the Enhanced Hybrid YOLO12 as the top performer in terms of overall detection quality. While YOLO11_CBAM achieves the highest precision (0.943) and YOLO11-BRA-Net leads in recall (0.907), the Enhanced Hybrid YOLO12 strikes a strong balance with high precision (0.942) and competitive recall (0.876). Most notably, it achieves the highest mAP50-95 score (0.735), indicating superior detection consistency across varying IoU thresholds. This suggests that the Enhanced Hybrid YOLO12 model offers the most robust and generalizable performance in complex agricultural environments, outperforming both baseline and improved variants in multi-metric evaluation.

**Fig. 6:**
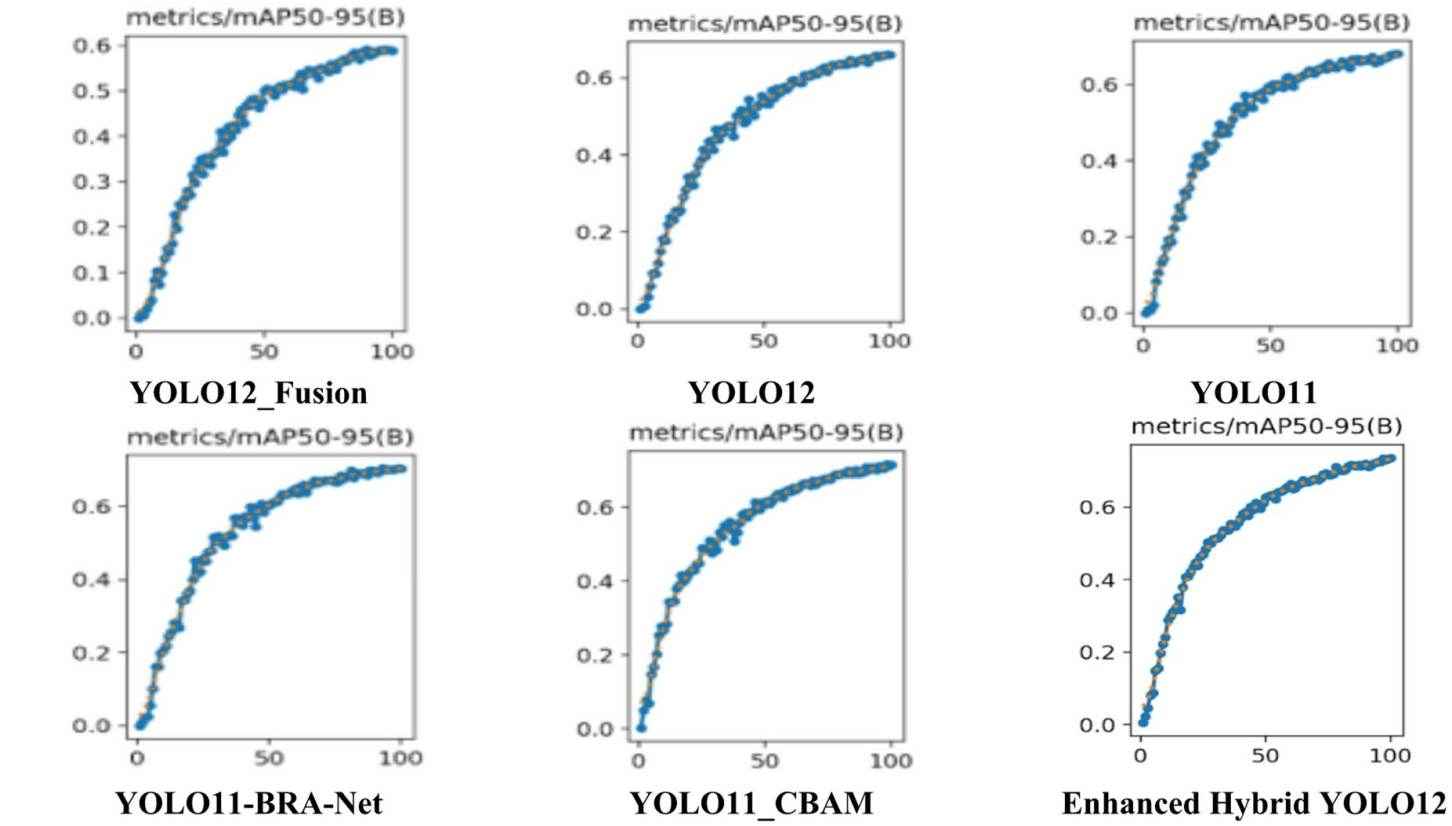
mAP50-95 for enhanced hybrid YOLO12 model versus other models.

**Fig. 7:**
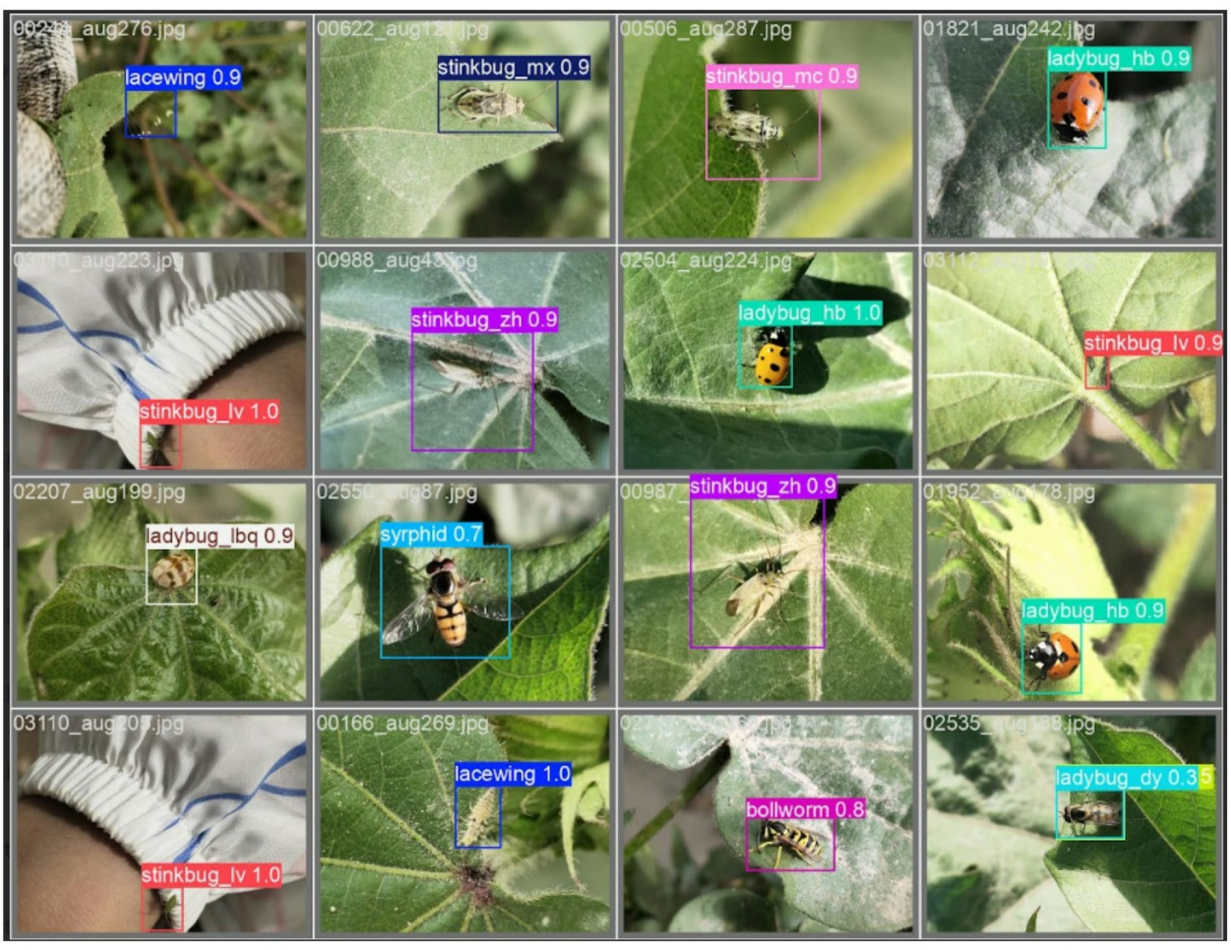
Samples from the detected cotton insects using the enhanced hybrid YOLO12 model.

Figure 6 illustrates the benefits of the Enhanced Hybrid YOLO12 model. It offers excellent detection accuracy compared to other state-of-the-art models in terms of mAP50-95. This metric offers comprehensive performance evaluation compared to traditional metrics such as mAP50 or mAP75. It evaluates the model at multiple values, from 50% to 95% in 5% increments, making it a more reliable detection metric, ensuring that the model detects objects and provides precise detection boxes. Figure 7 confirms this by showing that the proposed model precisely detects cotton insects with excellent accuracy and clear location of objects and overcomes competitors through mAP50-95 analysis and quality detection, which is highly effective for insect detection applications.

**Table 3:** Comparison among the enhanced hybrid YOLO12 model versus the other models.

Model	Precision	Recall	mAP50	mAP50-95
YOLO12_Fusion	0.865	0.833	0.866	0.592
YOLO12	0.925	0.848	0.913	0.662
YOLO11	0.914	0.878	0.927	0.679
YOLO11-BRA-Net	0.932	**0.907**	0.942	0.705
YOLO11_CBAM	**0.943**	0.906	**0.946**	0.716
**Enhanced hybrid YOLO12**	0.942	0.876	0.945	**0.735**

Significant values are in bold.

**Fig. 8:**
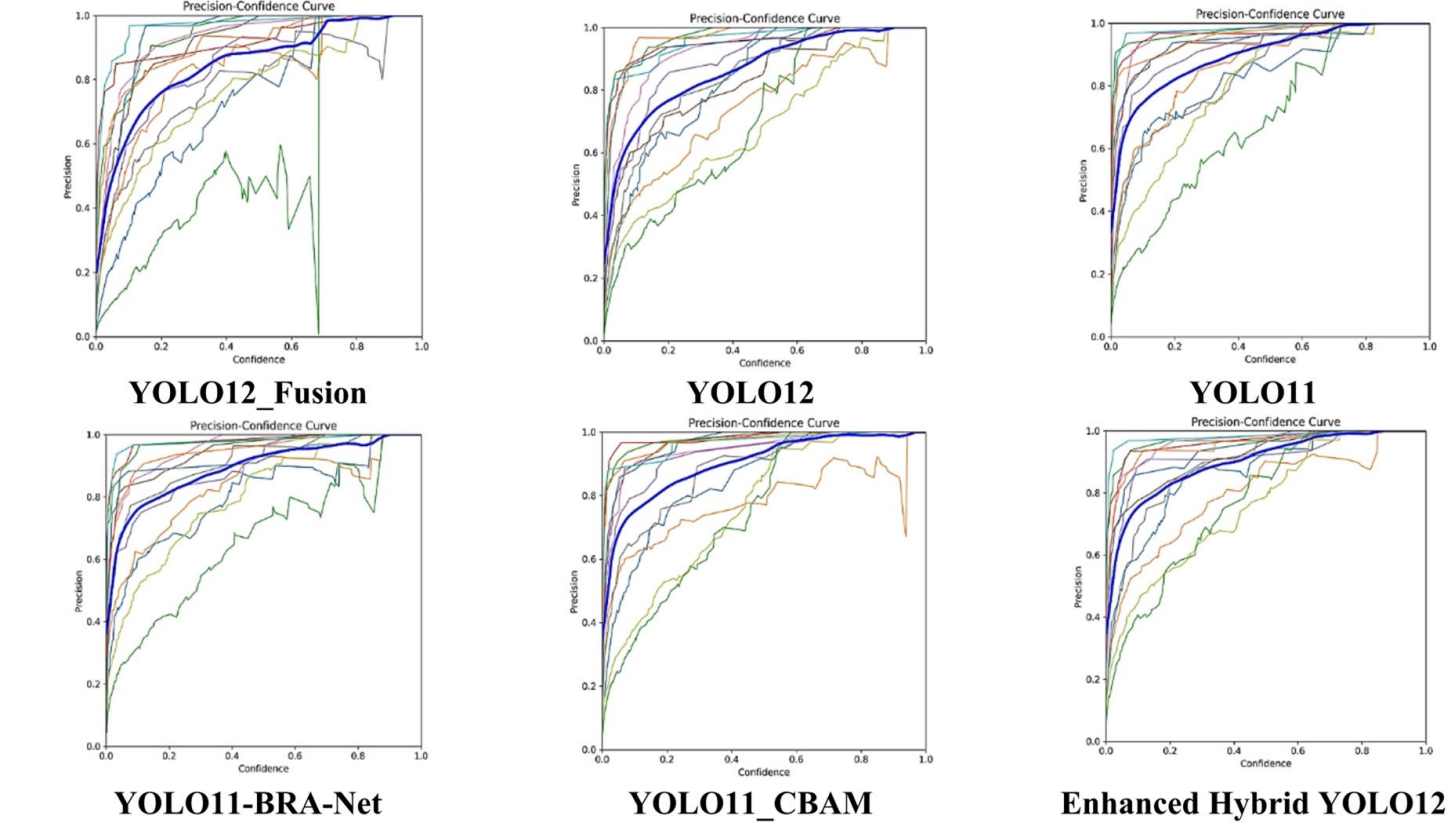
Precision-confidence curves of the enhanced hybrid YOLO12 model versus other models.

On the other hand, Fig. 8 presents the Precision-Confidence curve analysis presenting the performance of several state-of-the-art models with the proposed Hybrid YOLO12 model exhibiting enhanced accuracy at different confidence levels. The blue curve denoting “all classes” representing the thirteen cotton insects’ classes in each graph indicates that the Enhanced Hybrid YOLO12 model consistently exhibits superior precision across various confidence levels in comparison to other models, including YOLO11, YOLO12, and their variants (YOLO12_Fusion, YOLO11_CBAM, and YOLO11-BRA-Net). The Enhanced Hybrid YOLO12 model demonstrates a steeper rise and smoother progression signifying a reduction in false positives and enhanced classification reliability. The other models especially YOLO11 and its derivatives exhibit greater fluctuations indicating reduced stability and precision at specific confidence levels. The results support that the Hybrid YOLO12 model effectively balances precision and confidence, making it more accurate in detecting cotton insects relative to the other assessed models.

**Fig. 9:**
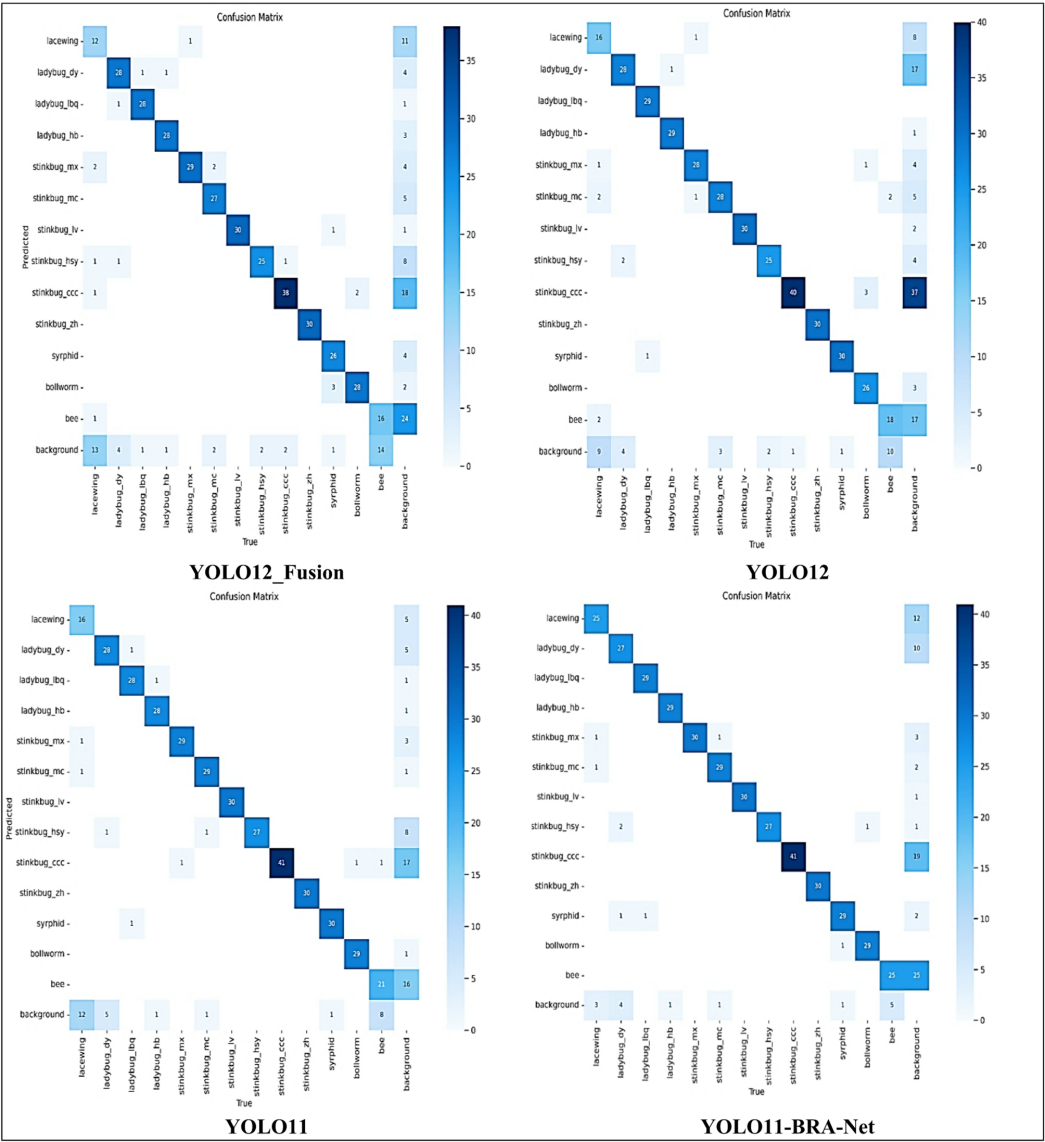

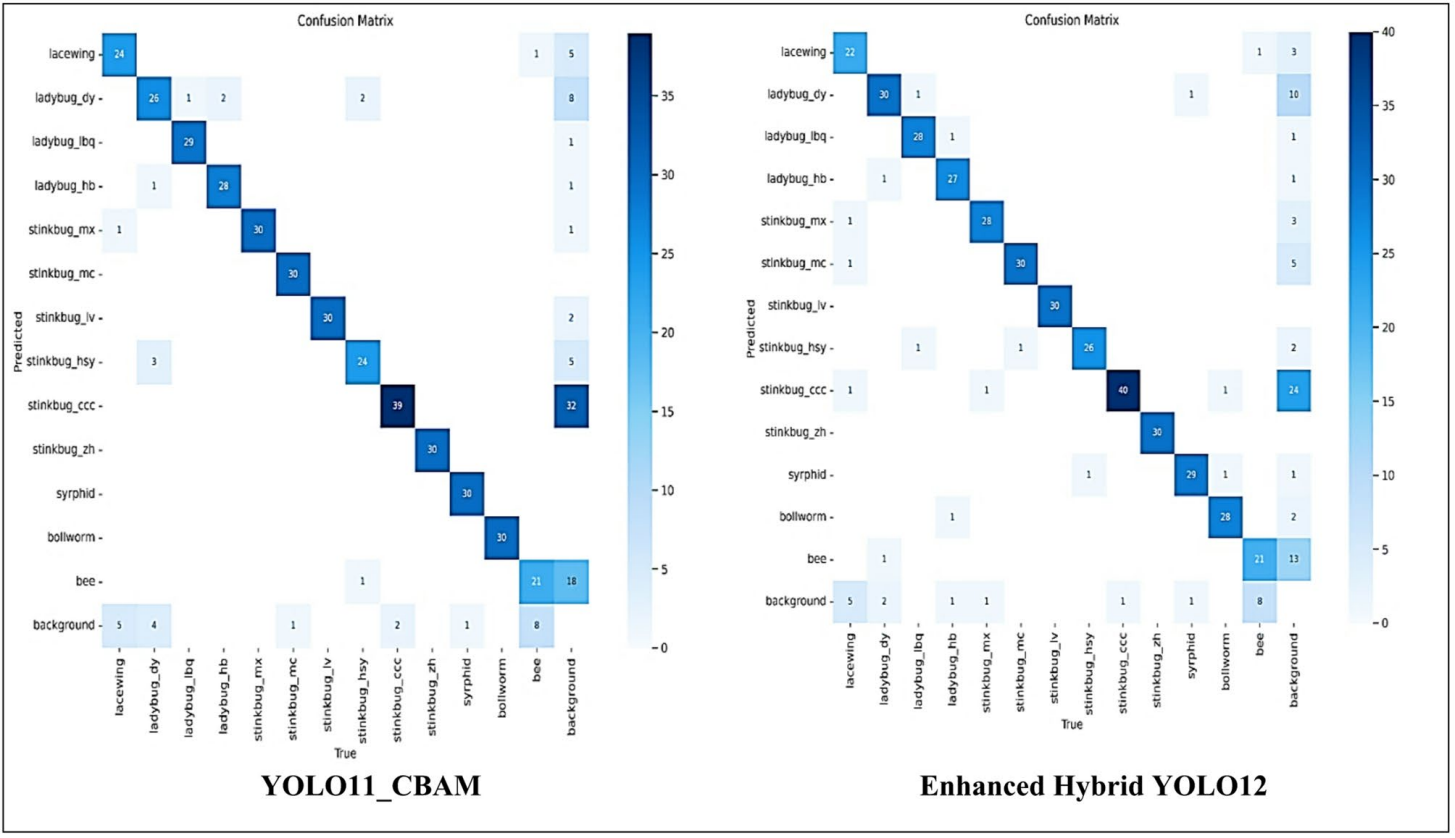
Confusion matrices of the enhanced hybrid YOLO12 model versus other models.

Furthermore, the confusion matrices in Fig. 9 illustrates the classification performance of several state-of-the-art models with the Enhanced Hybrid YOLO12 model, which shows increased accuracy in insect detection Darker diagonal slices in the matrix of the confusion of the Enhanced Hybrid YOLO12 model indicate higher actual positive rates compared to other models such as YOLO11, YOLO12 and their variants (YOLO12_Fusion, YOLO11_CBAM, and YOLO11-BRA-Net). On the other hand, alternative models show that it increases beyond the diagonal incorrect classifications, indicating a greater incidence of false positives and false negatives. The Enhanced Hybrid YOLO12 model significantly reduces the wrong classification, especially for analogous insects, resulting in more reliable predictions.

**Table 4:** Computational efficiency analysis of the enhanced hybrid YOLO12 versus other models.

Model	Total inference time per image (ms)	GFLOPs	Memory Consumption (GB)	Model Size (MB)
YOLO 12_Fusion	6.2	6.6	6.22	5.6
YOLO12	3.9	6.3	6.19	5.5
YOLO11	2.3	6.3	5.08	5.5
YOLO11-BRA-Net	5.7	11.1	5.87	5.9
YOLO11_CBAM	3.6	6.3	1.43	5.5
Enhanced hybrid YOLO12	6.4	42.8	3.21	31.4

### Computational efficiency analysis

Evaluating computing efficiency is necessary for analyzing DL models, especially for real-time applications such as insect detection^57^. Table 4 illustrates that, despite the Enhanced Hybrid YOLO12 model incurring greater computational costs for inference time, GFLOPs, memory usage, and model size relative to other models, it attains the highest detection accuracy with a mAP50-95 of 0.735. The exceptional accuracy and strong performance metrics of the Hybrid YOLO12 establish it as the optimal model for balancing detection precision and processing efficiency making it particularly appropriate for applications requiring high precision.

While YOLO11_CBAM achieves a little higher precision of 0.943, the Enhanced Hybrid YOLO12 model exhibits superior overall performance by maintaining a strong balance between precision at 0.942 and recall at 0.876. This balance yields the highest mAP50-95 score (0.735) among all models showing its resilience over diverse IoU thresholds. The significant improvement in detection accuracy validates the computational cost indicated in Table 4, as the model effectively trades increased complexity for better detection capability. The Enhanced Hybrid YOLO12 model with its optimal combination of efficiency and performance is an exceptionally effective choice for accurate insect detection while ensuring reasonable computational costs. However, the recall of Enhanced Hybrid YOLO12 model is slightly lower than that of YOLO11. This can be attributed primarily to stricter detection thresholds, and dataset imbalance. Specifically, our model emphasizes reducing false positives and improving localization accuracy thus achieving higher overall robustness, which leads to improved precision and F1-score at the cost of a marginal decrease in recall. In the context of precision agriculture, false positive tests can result in unnecessary pesticide use, leading to increased operating costs and negative environmental impacts. Therefore, it is often more appropriate to prioritize precision over recall for sustainable pest management systems. Furthermore, previous studies have shown that the relative importance of evaluation metrics is application dependent and should be guided by the costs associated with different error types^58,59^.

### Time complexity analysis

The time complexity of the Enhanced Hybrid YOLO12 model can be systematically analyzed using Big O Notation^58^. The time complexity using the Big O Notation of each component of the model is calculated and the model’s overall time complexity is determined as follows^60^:

1. Backbone component: the backbone component consists of multiple convolutional layers, including standard convolutions and modules C3K2 and SPPF, which integrate several blocks.

- Conv Layers (Convolutional Layers): Each standard convolution operation has a complexity of O(n²·k²·c_input·c_output) where n² is the spatial dimension, k² is the kernel size, c_input and c_output are input/output channels. For multiple convolutional layers: O(L_conv·n²·k²·c²), where L_conv is the number of layers.
- C3K2 Layers (Conv ×3 with kernel size 2): These involve 1 × 1 convolutions, split operations, and transitions. Time complexity is O(n²·c²) for the 1 × 1 convolutions + O(n²·4·c²) for kernel size 2 operations.
- SPPF: Max pooling operations with different kernel sizes are implemented to the input feature vector and then feeding them through the convolution layers. Time complexity is O(n²·c) for pooling + O(n²·k²·c²) for subsequent convolutions.

2. Neck component: the neck component involves several key modules including C2F, A2C2F, Up sample, Concat, and SPPF.

- C2F modules, being based on convolutional layers, contribute O(n²·k²·c²).
- The A2C2F module, integrating channel and spatial attention with C2F blocks, has a complexity of O(n²·k²·c²+ c²+ n²),
- Up sample operations have a complexity of O(n²⋅c).
- Concat operations have a complexity of O(n²⋅c).
- SPPF, which applies max pooling operations followed by convolutions, has a complexity of O(n²·c) for pooling + O(n²·k²·c²) for subsequent convolutions.

3. Head component: the head component has the final detection layers with convolutions. The complexity is O(n²·k²· c²).
4. Total time complexity: to obtain the overall model’s complexity, we sum the dominant terms from each component as O(L_backbon·n²·k²·c² + L_neck·n²·k²·c² + L_head·n²k²·c²). This can be simplified to O[(L_backbone + L_neck + L_head)·n²·k²·c²]. Since the architectural constants (i.e. number of layers) can be considered fixed for a specific model implementation, the final time complexity is O(n²·k²·c²).

This complexity is consistent with the model’s reported computational efficiency metrics: “GFLOPS of 42.8, memory consumption of 3.21 GB, model size of 31.4 MB, and inference time of 6.4 ms per image processing”, enabling it to perform real-time cotton insect detection.

While the hybrid model introduces a slight increase in inference time due to its structure which includes attention blocks to help in capturing the features of the small objects (i.e. insects), it achieves a better balance between efficiency and accuracy. The performance improvements, though moderate in magnitude, are consistent and meaningful for insect detection and classification. Our aim is to optimize the model’s robustness and accuracy without a significant computational penalty rather than to outperform all models in raw speed.

### Ablation study

To understand the effect of each proposed component, we carried out an ablation study on the C3K2, SPPF, and A2C2F modules which were used to enhance the performance of the Enhanced Hybrid YOLO12 model. In each experiment, one module was removed while the rest of the network remained unchanged. The overall performance and computational cost of the different variants are listed in Table 5, and the class-wise mAP50–95 for representative insect species is shown in Table 6.

The complete model that includes C3K2, SPPF, and A2C2F serves as our reference. It reaches a precision of 0.942, recall of 0.876, mAP50 of 0.945, and mAP50–95 of 0.735 with 15.32 M parameters and 42.6 GFLOPs. At the class level, it provides strong localization performance for most species, for example 0.870 for *stinkbug_lv*, 0.896 for *stinkbug_zh*, 0.925 for *bollworm*, and 0.812 for *syrphid*, while more difficult categories such as *lacewing* and *bee* still obtain 0.543 and 0.471, respectively.

When C3K2 is removed, the network becomes much lighter (4.57 M parameters, 14.3 GFLOPs), but this comes at a clear cost in accuracy. The global mAP50–95 drops from 0.735 to 0.692 and mAP50 falls to 0.930. The per-class results show that several insects are particularly affected: *bee* drops to 0.280, *stinkbug_mx* to 0.692, *stinkbug_lv* to 0.801, and *lacewing* to 0.483. These losses suggest that C3K2 is especially important for capturing fine local structure and for handling small or texture-rich targets. A few classes, such as *ladybug_dy* (0.608) and *stinkbug_ccc* (0.714), remain relatively stable, indicating that not all categories are equally dependent on this module.

The effect of removing SPPF is less dramatic but still substantial. This variant keeps the same complexity as the full model (15.32 M parameters, 42.6 GFLOPs) and even slightly increases precision to 0.951, yet mAP50 and mAP50–95 decrease to 0.936 and 0.719. In the class-wise results, most species show small drops compared with the full configuration, for example *lacewing* (0.504 vs. 0.543), *stinkbug_mx* (0.768 vs. 0.780), and *syrphid* (0.796 vs. 0.812). The performance on *bee* is clearly weaker (0.343), which points to the benefit of SPPF in providing global context when the background is cluttered or the object boundary is ambiguous. At the same time, some species such as *stinkbug_hsy* (0.620) and *stinkbug_ccc* (0.713) are close to or slightly above the full model, suggesting that not all classes rely equally on this type of context aggregation.

Removing A2C2F yields a more compact model (13.9 M parameters, 37.5 GFLOPs) while preserving most of the performance: precision is 0.928, recall 0.887, mAP50 is 0.939, and mAP50–95 is 0.725. For many classes, this variant is comparable to or better than the other ablations; for instance, *bollworm* reaches 0.937 and *stinkbug_lv* 0.868. However, for categories with more delicate shapes or boundaries, such as *lacewing* (0.511) and *bee* (0.418), the full model still performs better. This pattern suggests that A2C2F mainly helps to refine multi-scale attention and boundary localization, improving the reliability of predictions rather than giving large gains in the global metrics.

Taken together, these results indicate that the three modules are complementary. C3K2 is the key driver for fine-grained feature extraction and small-object discrimination, SPPF stabilizes performance by incorporating global spatial context, and A2C2F sharpens attention and boundary quality at relatively low extra cost. The full configuration, which keeps all three, therefore offers the most favourable balance between accuracy and efficiency and is used as the final model.

**Table 5:** Ablation study of C3K2, SPPF, and A2C2F modules: comparison of model size, computational cost, and detection performance (P, R, mAP50, mAP50–95).

Model variant	#Params (M)	GFLOPs	Precision	Recall	mAP50	mAP50–95
Full (C3K2 + SPPF + A2C2F)	15.32	42.6	0.942	0.876	0.945	0.735
Ablation (SPPF)	15.32	42.6	0.951	0.875	0.936	0.719
Ablation (A2C2F)	13.9	37.5	0.928	0.887	0.939	0.725
Ablation (C3K2)	4.57	14.3	0.945	0.867	0.930	0.692

**Table 6:** Class-wise mAP50–95 comparison for the full model and ablation variants (SPPF, A2C2F, C3K2).

Class	Full model (C3K2 + SPPF + A2C2F)	Ablation (SPPF)	Ablation (A2C2F)	Ablation (C3K2)
lacewing	0.543	0.504	0.511	0.483
ladybug_dy	0.584	0.567	0.587	0.608
ladybug_lbq	0.808	0.803	0.790	0.790
ladybug_hb	0.805	0.795	0.804	0.765
stinkbug_mx	0.780	0.768	0.770	0.692
stinkbug_mc	0.793	0.786	0.774	0.767
stinkbug_lv	0.870	0.858	0.868	0.801
stinkbug_hsy	0.563	0.620	0.625	0.557
stinkbug_ccc	0.706	0.713	0.688	0.714
stinkbug_zh	0.896	0.896	0.863	0.844
syrphid	0.812	0.796	0.795	0.774
bollworm	0.925	0.903	0.937	0.918
bee	0.471	0.343	0.418	0.280

### Environmental and economic benefits of the proposed model

The Enhanced hybrid YOLO12 provides substantial environmental and economic benefits through accurate identification of cotton insects in real time. It alleviates the abuse of pesticides supporting sustainable agriculture and ensures biodiversity. It reduces labour costs by automating insects monitoring, increasing the efficiency of resources and increasing the production and quality of crops, thereby maximizing the profitability of farmers. Its computing efficiency ensures minimal energy utilization, which is suitable for real-time use in intelligent agricultural systems. Moreover, thanks to its scalability and the use of publicly accessible statistics, it is also available for both large and small farmers who support extensive acceptance in precise agriculture.

## Conclusion and future work

This study presents an investigation of using multiple YOLO models in the detection and classification of cotton insects using a new dataset existing in the literature. Among these models, the Enhanced Hybrid YOLO12, a novel variant of the YOLO12 framework proved to be the most competing model among the 6 investigated models. By incorporating optimized feature extraction and attention mechanisms, the model achieves superior detection performance, surpassing both standard YOLO11 and YOLO12 and our custom variants and delivering the highest mAP50-95 while maintaining strong precision and recall. Beyond accuracy, Enhanced YOLO12 demonstrates a favourable balance between detection performance and computational efficiency, making it a reliable and scalable solution for precision agriculture. Although inference time is slightly higher than the standard YOLO12, the model remains viable for real-time field applications. This work underscores the transformative role of deep learning in sustainable farming, enabling automated, accurate, and scalable insect management strategies.

Although the Enhanced Hybrid YOLO12 model shows strong detection accuracy, implementation on low-power edge devices may be limited by its slightly longer inference time and dependency on high-performance hardware. Furthermore, the model’s assessment is limited to a particular dataset and bug species, which could limit its applicability to more expansive agricultural settings and unidentified species.

Looking ahead, future research will focus on optimizing Enhanced Hybrid YOLO12 for edge-device deployment, expanding the dataset to include a broader range of insect species, and integrating multispectral imaging to further improve detection under complex environmental conditions.

## Data Availability

The dataset is publicly available and can be accessed at the following link: [https://www.scidb.cn/en/detail? dataSetId=3f36bce8e41849a6a33e34fb0f8ae581](https:/www.scidb.cn/en/detail? dataSetId=3f36bce8e41849a6a33e34fb0f8ae581).

## Abbreviations

A2C2F: Attention mechanism with C2F block
AI: Artificial intelligence
AELGNet: Attention-based enhanced local and global features network
BERT-ResNet-PSO: Bidirectional encoder representations from transformers-residual network-particle swarm optimization
BiFormerAF: BiFormer attention fusion
BRA: Bi-level routing attention
C2F: Cross stage partial bottleneck with 2 convolutional layers
C2PSA: Convolutional block with parallel spatial attention
C3K: Conv ×3 with kernel
C3K2: Conv ×3 with kernel size 2
CAM: Channel attention module
CBAM: Convolutional block attention module
CFNet-VoVGCSP-LSKNet-YOLOv8s: Cross-feature network- VoVNet with ghost convolutional structure and spatial pyramid- large kernel attention network- you only look once version 8
CNN: Convolutional neural network
COLAB: Google collaboratory
C2PSA: Cross-stage partial self-attention
CSP: Cross stage partial
DenseNet121: Densely connected convolutional network with 121 layers
DL: Deep learning
ECENet: EfficientNet model
InceptionResNetV2: Inception residual network version 2
F-measure: Fisher score-measure
FLOPS: Floating point operations per second
FM: Feature map
FM-SR: Feature map-super resolution
FN: False negative
FP: False positive
GCSP: Ghost CSP
GFLOPS: Giga floating-point operations per second
mAP: Mean average precision
ML: Machine learning
MSP2P: Multi-scale patch-to-patch
ResNet50: Residual network with 50 layers
SAM: Spatial attention module
SPPF: Spatial pyramid pooling fast
SpemNet: Stacking patch embedding network
SRNet-YOLO: Super-resolution network-you only look once
TN: True negative
TP: True positive
TXT: Text format
ViT: Vision transformer
VGG16: Visual geometry group neural network with 16 layers
XML: Extensible markup language
YOLO: You only look once
YOLOv8-MDN-Tiny: YOLO version 8 with mixed density network-tiny
YOLO11: YOLO version 11
YOLO11-BRA-Net: YOLO11 with bi-level routing attention network
YOLO11_CBAM: YOLO11 with convolutional block attention module
YOLO12: YOLO version 12
YOLO12_Fusion: YOLO12_Fusion

## Code availability

The custom code used in this study to generate and analyse the results is publicly available in a GitHub repository at https://github.com/DrDinaSaif/Enhanced-YOLO-12.
